# Improvement in Long-Term Memory following Chronic Administration of *Eryngium planum* Root Extract in Scopolamine Model: Behavioral and Molecular Study

**DOI:** 10.1155/2015/145140

**Published:** 2015-09-21

**Authors:** Marcin Ozarowski, Barbara Thiem, Przemyslaw L. Mikolajczak, Anna Piasecka, Piotr Kachlicki, Michal Szulc, Ewa Kaminska, Anna Bogacz, Radoslaw Kujawski, Joanna Bartkowiak-Wieczorek, Malgorzata Kujawska, Jadwiga Jodynis-Liebert, Jaromir Budzianowski, Izabela Kędziora, Agnieszka Seremak-Mrozikiewicz, Boguslaw Czerny, Teresa Bobkiewicz-Kozłowska

**Affiliations:** ^1^Department of Pharmaceutical Botany and Plant Biotechnology, Poznan University of Medical Sciences, Sw. Marii Magdaleny 14, 61-861 Poznan, Poland; ^2^Department of Pharmacology and Phytochemistry, Institute of Natural Fibers and Medicinal Plants, Libelta 27, 61-707 Poznan, Poland; ^3^Department of Pharmacology, University of Medical Sciences, Rokietnicka 5a, 60-806 Poznan, Poland; ^4^Department of Pathogen Genetics and Plant Resistance, Metabolomics Team, Institute of Plant Genetics of the Polish Academy of Science, Strzeszynska 34, 60-479 Poznan, Poland; ^5^Laboratory of Experimental Pharmacogenetics, Department of Clinical Pharmacy and Biopharmacy, University of Medical Sciences, Sw. Marii Magdaleny 14, 61-861 Poznan, Poland; ^6^Department of Toxicology, Poznan University of Medical Sciences, Dojazd 30, 60-631 Poznan, Poland; ^7^Division of Perinatology and Women's Diseases, Poznan University of Medical Sciences, Polna 33, 60-535 Poznan, Poland; ^8^Laboratory of Molecular Biology, Poznan University of Medical Sciences, Polna 33, 60-535 Poznan, Poland; ^9^Department of General Pharmacology and Pharmacoeconomics, Pomeranian Medical University, Zolnierska 48, 70-204 Szczecin, Poland; ^10^Department of Stem Cells and Regenerative Medicine, Institute of Natural Fibres and Medicinal Plants, Libelta 27, 61-707 Poznan, Poland

## Abstract

*Eryngium planum* L. (EP) is as a rare medicinal plant with a lot of potentials as pharmaceutical crops. The aim of our study was to assess the effect of subchronic (28-fold) administration of a 70% ethanol extract of EP roots (200 mg/kg, p.o.) on behavioral and cognitive responses in Wistar rats linked with acetylcholinesterase (AChE), butyrylcholinesterase (BuChE), and beta-secretase (BACE-1) mRNA levels and AChE and BuChE activities in the hippocampus and frontal cortex. On the last day of experiment, 30 min after the last dose of EP or Huperzine A (HU), scopolamine (SC) was given at a dose of 0.5 mg/kg b.w. intraperitoneally. The results of a passive avoidance test showed an improvement in long-term memory produced by the EP extract in both scopolamine-induced rats and control group. EP caused an insignificant inhibition of AChE and BuChE activities in the frontal cortex and the hippocampus. EP decreased mRNA AChE, BuChE, and BACE-1 levels, especially in the cortex. Our results suggest that the EP extract led to the improvement of the long-term memory in rats coupled with total saponin content. The mechanism of EP action is probably complicated, since HPLC-MS analysis showed 64 chemical compounds (phenolics, saponins) in the extract of EP roots.

## 1. Introduction

Nowadays, a global trend for discovery and development of new drugs from natural sources to prevent or slow the progression of neurodegenerative diseases (e.g., Alzheimer's and Parkinson's diseases) is clearly visible [1, 2]. Numerous studies on modern pharmacology focused on* in vitro* screening for acetylcholinesterase inhibitory activity [3–6], which contributes to the development of Alzheimer's disease (AD).

AD is a progressive process associated with memory loss and deficits in cognitive and memory functions. Most of the currently available drug therapies are based on the “cholinergic hypothesis,” which suggests that AD begins as a deficiency in the production of acetylcholine neurotransmitter [7]. Therefore, acetylcholinesterase (AChE) and butyrylcholinesterase (BuChE) enzyme coinhibition is considered to be a potential alternative for the treatment of AD [8]. The pathogenesis of AD is associated with several biological risk factors: gene mutation, beta-amyloid deposition, oxidative stress, inflammatory process, and dysfunction of the cholinergic system in the brain [9]. The above-mentioned studies are also a part of the search for new neuroprotective natural origin drug candidates for AD treatment. On the other hand, plant extracts containing various biologically active compounds may exert a number of pleiotropic effects in CNS, not only by elevation of acetylcholine concentration in the synaptic cleft, but also by decreasing beta-amyloid deposition and due to their antioxidative properties. Recently, herbal medicines have received much attention and are recommended as a natural way of maintaining or improving mental and physical health. In traditional herbal medicine, numerous plant extracts have been used to treat age-related cognitive disorders [1, 10, 11]. Some authors demonstrated that certain herbs may become important sources of new therapeutic medicines for dementia and allow us to discover drug candidates against the onset of AD. For example, Huperzine A, isolated from Chinese herbal moss* Huperzia serrata *[12], and galanthamine, isolated from* Ungernia* sp. [13], are commonly used as cholinesterase inhibitors. Experimental reports suggest that some plant extracts and isolated chemical compounds (e.g., luteolin and myricetin) could have neuroprotective effects against beta-amyloid* via* inhibition of beta-secretase (BACE-1) [14, 15].

*Eryngium planum *L. (Apiaceae, subfamily Saniculoidae) is one of the most promising medicinal plants used in traditional medicine in Europe. Some* Eryngium *species are cultivated as ornamental, vegetable, or medicinal crops for folk uses. With increasing chemical and biological investigations and ethnobotanical studies,* Eryngium *has shown its potential as pharmaceutical crops [16]. Phytochemical investigations of* Eryngium* species have yielded the presence of phenolic acids [17], flavonoids, namely, kaempferol and quercetin glycosides [18, 19], triterpenoid saponins [20], coumarin derivatives [21], essential oils [22], and acetylenes [22, 23]. Complex saponins are the main group of secondary metabolites accumulated in the root of EP [16]. Moreover,* Eryngium* species possess several pharmacological properties, for example, diuretic, antidiabetic, expectorant, spasmolytic, anti-inflammatory, antinociceptive, hemolytic, and antimycotic [24–26], which depend mainly on their saponin content, but the presence of phenolic acids, polyacetylenes, and flavonoids is also important for their usage in European traditional medicine [16].

## 2. Objectives

The aim of our study was to evaluate the influence of subchronic (28-fold) administration of the* E. planum *root extract on scopolamine (SC) impaired memory in Wistar rats. Furthermore, assessment of acetylcholinesterase (AChE) and butyrylcholinesterase (BuChE) activity in the hippocampus and the frontal cortex was investigated. Moreover, gene expression levels for AChE, BuChE, and BACE-1 in the hippocampus and the frontal cortex were determined.

## 3. Material and Methods

### 3.1. Plant Material

The plant material, roots of* Eryngium planum* L. (EP), was collected from a natural site in Poland (Kujawy region). The plant material was identified by Professor Janina Borysiak of Adam Mickiewicz University in Poznan. The voucher specimen (number 6725) is deposited in the Herbarium of Medicinal Plant Garden in the Institute of Natural Fibers and Medicinal Plants, Poznan, Poland.

### 3.2. Chemicals and Drugs

All reagents for TLC and HPLC analysis, scopolamine hydrobromide trihydrate (SC), and reagents for biochemical analyses were purchased from Sigma-Aldrich (Poland). Huperzine A (HU) was obtained from Enzo Life Sciences AG (Alexis Corporation, Biomibo Distribution, Poland). Chemicals for gene expression analysis were obtained from Roche Diagnostic and ALAB (Poland). All chemicals and drugs were* ex tempore* prepared on the day of the experiment.

### 3.3. Preparation of the Extract and Phytochemical Analysis

Dried and powdered root of* E. planum* (1017 g) was extracted with 70% ethanol using the percolation method (24 h) at room temperature (22°C ± 1) and filtered. The extract was concentrated using rotary vacuum evaporator to give a dry residue. The yield of the extract was 440.0 g. A small portion of the extract (1 mL) was analyzed and profiled using HPTLC and HPLC-DAD, according to the methods applied in earlier studies [27], to determine the presence of the compounds. Moreover, yield and composition of essential oils from the roots were determined by GC-FID-MS and the content of falcarinol in the ethanol extract was evaluated as described earlier [22]. Dried and powdered roots of* E. planum* (351.0 g) were extracted with boiling 70% ethanol (4 × 3 L). The combined extracts were evaporated to give a dry extract (145.5 g), a portion of which (136.0 g) was separated by column chromatography on polyamide using water, 100% methanol, and methanol with 0.1% ammonia for elution. Column fractions were combined according to the results of TLC examination (see Phytochemical Screening) to give sugar, saponin, and phenolic acid fractions.

### 3.4. Phytochemical Screening

#### 3.4.1. TLC (Thin Layer Chromatography)

Equal portions of each extract or fraction (0.1 g) were dissolved in 1 mL of 70% ethanol. The presence of phenolic acids and flavonoids was analyzed on cellulose or silica gel HPTLC plates (Merck), developed with ethyl acetate-acetic acid water (8 : 1 : 1). The developed plates were viewed under UV_254_ and UV_366_ nm light before and after spraying with 0.1% NA (2-aminoethanol diphenyl borate) in ethanol. Brown bands changing to yellow or orange fluorescence were considered as those of flavonoids, while blue bands changing to strong blue or blue-yellow fluorescence under UV were considered as those corresponding to phenolic acids. Coumarins were recognized by strong blue fluorescence without staining. Analysis for saponins was performed by TLC on silica gel F plates (Merck), eluted with 1-butanol-acetic acid water (4 : 1 : 5) (organic phase), and detection was carried out by spraying with vanillin-sulfuric acid or Liebermann-Burchard reagent, followed by heating for 5 min at 100°C. Saponins appeared as violet-pink bands in daylight.

#### 3.4.2. HPLC (High Performance Liquid Chromatography)

Identification of secondary metabolites present in the roots of* E. planum *was performed using the HPLC-MS system consisting of Agilent 1100 HPLC instrument with a photodiode-array detector PDAe*λ* (Palo Alto, CA, USA) and Esquire 3000 ion trap mass spectrometer (Bruker Daltonics, Bremen, Germany) with the XBridge C18 column (150 × 2.1 mm, 3.5 *μ*m particle size) and the MS^*n*^ spectra were recorded in the negative and positive ion modes using a previously published approach [28, 29]. The elution was conducted with water containing 0.1% formic acid (solvent A) and acetonitrile (solvent B). The gradient elution was started at 10% of B and linearly changed to 25% of B in 25 min and to 98% in 46 min of B over 10 min, followed by return to stationary conditions and reequilibration for 10 min. Moreover, the UHPLC-MS/MS was applied for the quantitative analysis of phenolic compounds (flavonoids and phenolic acids) and for the analysis of triterpenoid saponins. Methods and results were described previously [30].

#### 3.4.3. Determination of Total Phenolic Compound in the Extract

The calculation of polyphenols to gallic acid (GA) was done using the Folin-Ciocalteu reagent with the modified spectrophotometric method described by Cheng et al. [31].

### 3.5. Rats

Experiments with rats were performed in accordance with the Polish governmental regulations (Dz. U. 05.33.289). The study was conducted in accordance with the ethical guidelines for investigations in conscious rats and the study protocol was approved by the Local Ethics Committee (64/2008). The experiments were performed on 6-week-old male Wistar rats, housed in controlled room temperature (20 ± 0.2°C) and humidity (65–75%) under a 12 h : 12 h light-dark cycle (lights on 7 a.m.). Rats were kept in groups (8–10 rats/group) in light plastic cages (60 × 40 × 40 cm) and given* ad libitum* access to standard laboratory chow (pellets, Labofeed B) and tap water.

### 3.6. Treatments

Rats were treated with the ethanol extract of EP roots at a dose of 200 mg/kg b.w., intragastrically (p.o.) (groups EP + H_2_O and EP + S) for 28 (28x) consecutive days. For comparative purposes, HU was administered at a dose of 0.5 mg/kg b.w. (p.o.) (groups HU + H_2_O and HU + SC) as a known acetylcholinesterase inhibitor. On the last day, 30 min after the last dose of EP, or HU, SC was given intraperitoneally (i.p.) at a dose of 0.5 mg/kg b.w. Control groups were treated with 0.5% methylcellulose (MC), and water for injection (H_2_O) was used as a vehicle for SC (groups MC + H_2_O and MC + SC). The EP extract was prepared* ex tempore* before administration and suspended in MC in concentrations of 20 mg/mL. On day 28 of the experiment, 1 h after the last dose, the rats were killed by decapitation. The hippocampus and a part of the frontal cortex were collected from rat brains. Tissue samples were then stored at −80°C until the measurement of AChE and BuChE activity or AChE, BuChE, and BACE-1 mRNA levels changes.

### 3.7. Cognitive and Behavioral Tests

The following cognitive and behavioral tests were used in our study: (1) sedative activity was assessed using a locomotor activity test, (2) motor coordination was evaluated using the “chimney” test, and (3) passive avoidance test and object recognition test were used for the assessment of long-term and short-term memory.

#### 3.7.1. Measurement of Locomotor Activity

Locomotor activity evaluation was performed with licensed activity meter (Activity Cage, Ugo Basile, Italy) by placing the rats in the center of the apparatus and recording their horizontal activity [29]. The obtained data were expressed as signals corresponding to rat movements for 5 min. Locomotor activity was measured 30 min after the administration of a single dose of scopolamine or the vehicle (H_2_O). Distracting factors (noise, presence of people, and presence of other rats) were reduced to the minimum.

#### 3.7.2. Measurement of Motor Coordination

Motor coordination was evaluated using the “chimney” test, described originally for mice [32]. Thirty min after scopolamine or vehicle injection, each rat was allowed to enter a glass laboratory cylinder, 500 mm in length and 80 mm in diameter, laid on its side. Upon reaching its bottom by the rat, the position of the cylinder was rapidly changed from horizontal to vertical, and the timer was started. The rat immediately began to move backwards. The timer was stopped after the rat left the cylinder and assumed a sitting position on the top of the vessel. The time of exit from the cylinder was accepted as a measure of motor coordination. Motor impairment was assessed as the inability of a rat to climb backwards up the tube within 60 s. The test was performed 30 min after the administration of a single dose of scopolamine or the vehicle.

#### 3.7.3. Passive Avoidance Test

Passive avoidance test was used in Wistar rats to assess long-term memory (effects on retrieval and memory consolidation) [33]. The test relies on the natural preference of rats for the dark. After 2 min of habituation to a dark compartment, the rat was placed on an illuminated platform and allowed to enter the dark compartment using licensed apparatus (Passive Avoidance System, step-through, Ugo Basile, Italy). Two more approach trials were allowed on the following day with a 2 min interval between them. At the end of the second trial, an unavoidable scrambled electric foot shock (500 *μ*A, AC, 3 s) was delivered through the grid floor of the dark compartment (learning trial). Retention of the passive avoidance response (latency) was tested 24 h later by placing the rat on the platform and measuring the latency in reentering the dark compartment against the arbitrary maximum time of 180 s. The test was performed 30 min after the administration of a single dose of scopolamine or the vehicle.

#### 3.7.4. Object Recognition Test

Object recognition test was used for assessing short-term memory [34]. The object recognition task took place in a 40 × 60 cm open box surrounded by 40 cm high walls made of plywood with a frontal glass wall. All rats were submitted to a habituation session during which they were allowed to freely explore the open field for 5 min. No objects were placed in the box during the habituation trial. On the day of the test, the rats were given an additional 3 min rehabituation period prior to commencing the test. The test was divided into three phases with two trials, the acquisition trial, the retention trial, and the intertrial interval of varying duration.

(i) Acquisition trial: in this first trial, the rats explored two identical objects (A1 and A2) positioned in two adjacent corners, 10 cm from the walls, for a period of 3 min.
(ii) Intertrial interval (ITI): the rats were returned to the home cage for 30 min.
(iii) Retention trial: in this second trial, the rats explored a familiar object (A^*∗*^), which is a duplicate of the objects from the acquisition trial (to minimize olfactory cues), and a novel object (B) for additional 3 min.

The objects were made of a biologically inert substance (plastic) and were chosen to enable ease of cleaning (10% alcohol) between subjects in an attempt to remove olfactory cues. Object exploration is defined as licking, sniffing, or touching the object whilst sniffing, but not leaning against, turning round, standing, or sitting on the object. Objects were of sufficient weight and were secured to the floor of the arena to ensure that they could not be knocked over or moved around by the rat. Exploration times of all objects were recorded using a stopwatch for subsequent statistical analysis. Time measured as the exploration behavior was used to calculate the memory discrimination index (OR), as reported by Blalock et al., OR = (B − A^*∗*^)/(B + A), where B was the time spent exploring the new object and A^*∗*^ was the time spent exploring the familiar object. Higher OR was considered to reflect greater memory ability [34]. The test was performed 30 min after the administration of a single dose of scopolamine or the vehicle.

#### 3.7.5. Acetylcholinesterase and Butyrylcholinesterase Activity Assay in Rat Brain

AChE and BuChE activities were investigated by modifying spectrophotometric Ellman's method according to Isomae et al. [35]. The activity of these enzymes was determined by measuring the formation of the yellow anion obtained from the reaction between Ellman's reagent and the thiocholine generated by enzymatic hydrolysis of acetylthiocholine iodide (ATCh) and butyrylthiocholine (BTCh), respectively (sample 0.1 mL, PBS 0.8 mL, DTNB 0.1 mL, ATCh 0.20 mL, and BTCh 0.20 mL). The biochemical assays were expressed as *μ*mol/min/mg protein by using the spectrophotometric method (412 nm).

#### 3.7.6. RNA Isolation and Reverse Transcription Reaction

Total RNA isolation from tissue homogenates of rat brains (frontal cortex, hippocampus) was carried out using TriPure Isolation Reagent (Roche), according to the manufacturer's protocol. The integrity of RNA was visually assessed electrophoretically and spectrophotometrically (BioPhotometer Eppendorf); 1 *μ*g of total RNA from all samples was used for reverse transcription into cDNA using Transcriptor First Strand Synthesis Kit (Roche), according to the manufacturer's protocol. Then, the samples were stored at −20°C or used directly for quantitative real-time PCR (qRT-PCR).

#### 3.7.7. Real-Time PCR mRNA Quantification

AChE, BChE, and BACE-1 mRNAs levels were analyzed by two-step quantitative real-time PCR (qRT-PCR), in a volume of 10 *μ*L reaction mixture, using relative quantification methodology with a LightCycler TM Instrument (Roche, Germany) and a LightCycler FastStart DNA Master SYBR Green I kit (Roche Applied Science), according to the manufacturer's instructions. All primer sequences were self-designed using Oligo 6.0 software (National Biosciences) and verified by the electrophoretic assessment and by a single temperature dissociation peak (melting curve analysis) of each cDNA amplification product. An GAPDH gene was used as a housekeeping gene (endogenous internal standard). Standard curves were prepared from dilution of cDNA and generated from a minimum of four data points for each quantified gene. All quantitative PCR reactions were repeated twice. Data were evaluated using LightCycler Run 4.5 software (Roche Applied Science). Each PCR run included a nontemplate control to detect potential contamination of reagents.

#### 3.7.8. Statistical Analysis

All values were expressed as means ± SEM. Statistical comparison of the results was carried out using one-way analysis of variance (ANOVA), followed by least significant difference post hoc test for detailed data analysis. A *P* value of <0.05 was considered as statistically significant.

## 4. Results

### 4.1. Phytochemical Profile of the Extract of* Eryngium planum* Root

**Figure 1:**
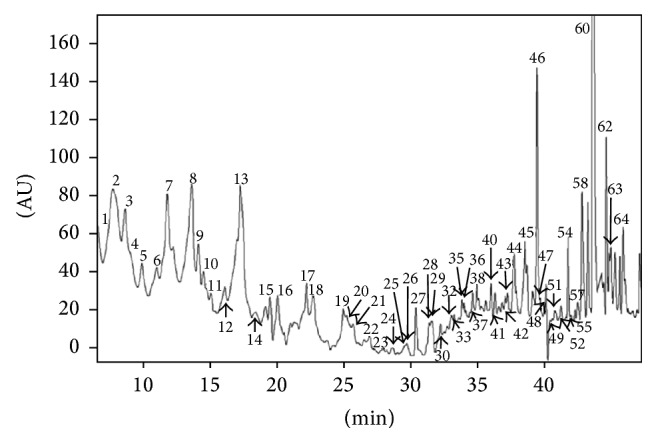
Chromatogram UV of EP extract obtained at 270 nm with peaks identified by HPLC-UV-MS.

Total polyphenols content of the EP extract determined with the use of Folin-Ciocalteu assay was 2.45 mg GA/g. It has been calculated that the content of rosmarinic acid is 0.055 mg g^−1^ d.w. [27]. Moreover, the main constituents of the studied EP root extract are triterpenoid saponins with barrigenol skeleton: M1: 3-O-*β*-D-glucopyranosyl-(1 → 2)-*β*-D-glucuronopyranosyl-22-O-angeloyl-R1-barrigenol, M2A: 3-O-*β*-D-glucopyranosyl-(1 → 2)-*β*-D-glucuronopyranosyl-21-O-acetyl, 22-O-angeloyl-R1-barrigenol, and M2B: 3-O-*β*-D-glucopyranosyl-(1 → 2)-*β*-D-glucuronopyranosyl-22-O-angeloyl-A1-barrigenol. The content of this fraction in the root extract was 5.138 mg g^−1^ d.w. [30]. HPLC-UV-MS showed that 64 various chemical compounds like triterpenoid saponins (36 compounds of unknown chemical structure), phenolic acids (*p*-coumaric acid and ferulic acid derivatives), polyphenolic acids (caffeic acid and rosmarinic acid derivatives), and others were determined in the EP extract (Table 1, Figure 1). The analysis revealed that* p*-coumaric, caffeic, and ferulic acids were identified in conjugation with glycosides (compounds numbers 3, 4, 6, and 7 in Table 1, Figure 1) in the EP extract. Glycosides of caffeic and ferulic acids were identified using mass spectrometric analysis in the negative ionization, whereas* p*-coumaric acid glycosides analysis could be identified in the positive ionization. Fragmentation of compound 6 in the negative ionization revealed sequential losses of 162 amu, followed by the loss of 342 amu (162 + 180), which indicated the presence of glucosyl and diglucosyl moieties bound to caffeic acid via* O*-glycosidic bonds. The losses of the 162 amu fragment constitute a typical fragmentation pattern of the glucose moiety in the negative ion mode, as was observed for compound 7 [36]. The loss of the whole glucose residue (180 amu) was observed in the positive ionization of compounds 3 and 4. The major ions observed in the spectra of these compounds in both ionization modes corresponded to the fragmentation of the particular hydroxycinnamic acids. Four glycosides and one glucuronide of rosmarinic acid (compounds numbers 8, 9, 10, 13, and 18) as well as rosmarinic acid alone (number 17) were identified in the negative ionization mode. Triterpenoid saponins constitute a large part of the detected metabolites (metabolites numbers 19–29 and 31–64). Only metabolites 26, 31, 40, 46, 45, 59, and 60 were precisely identified as derivatives of A1-barrigenol and R1-barrigenol, in accordance with the literature data presented in Table 1. The structures of the other saponins, in particular the places of substitution with glycosidic and acyl moieties, as well as the character of the substituents, could not be defined by mass spectrometry and require further detailed NMR analysis.

### 4.2. Cognitive and Behavioral Experiments

#### 4.2.1. Locomotor Activity

**Figure 2:**
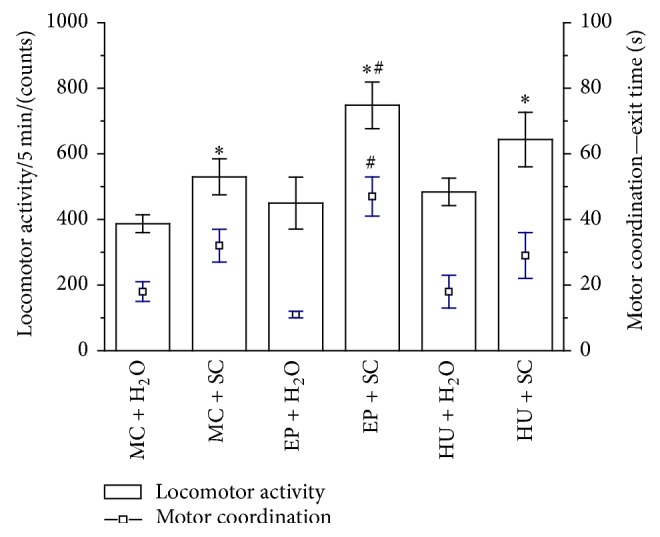
Effect of EP extract (200 mg/kg, p.o.) treatment on sedative activity and motor coordination in rats. Data are expressed as the means ± SEM. *n*: number of rats. MC + H_2_O: control rats (*n* = 20). MC + SC: scopolamine-treated group (0.5 mg/kg b.w., i.p.) (*n* = 20). EP + H_2_O: extract from root of* Eryngium planum* (*n* = 10). EP + SC: combined extract from root of* Eryngium planum *and scopolamine-treated rats (*n* = 9). HU + H_2_O: Huperzine A treated rats (0.5 mg/kg b.w. p.o.) (*n* = 10). HU + SC: combined Huperzine A and scopolamine-treated rats (*n* = 10). *∗*: versus MC + H_2_O, *P* < 0.05. #: versus MC + SC, *P* < 0.05.

A one-way ANOVA analysis revealed significant differences in the locomotor activity of rats, expressed as their horizontal spontaneous activity (*F*(5,73) = 4.89; *P* < 0.001; Figure 2). Detailed post hoc analysis showed that EP + H_2_O did not change the locomotor activity of rats, just as HU + H_2_O did not affect this activity as compared to the control group (MC + H_2_O). Stimulating effects in the locomotor activity of rats were observed after an acute S injection (MC + SC versus MC + H_2_O, *P* < 0.05; EP + SC versus MC + H_2_O, *P* < 0.05; HU + SC, *P* < 0.05). Moreover, higher activity was found in EP + SC rats in comparison to MC + SC rats (*P* < 0.05).

#### 4.2.2. Motor Coordination

A one-way ANOVA analysis revealed significant differences in motor coordination of rats, expressed as their exit time from the cylinder (*F*(5,73) = 5.90; *P* < 0.001; Figure 2). Detailed analysis showed that multiple administration of EP + H_2_O and HU + H_2_O did not significantly affect this paradigm. On the contrary, all SC-treated rats showed prolongation of the exit time, and the differences were statistically significant in control and EP-treated rats (*P* < 0.05) (MC + SC versus MC + H_2_O, *P* < 0.05; EP + SC versus MC + H_2_O, *P* < 0.05). Moreover, combination of SC and the EP extract led to more profound results because we observed the highest prolongation of the exit time as compared to MC + SC rats (*P* < 0.05), whereas for HU + SC rats the difference did not reach statistical significance.

#### 4.2.3. Long-Term Memory

**Figure 3:**
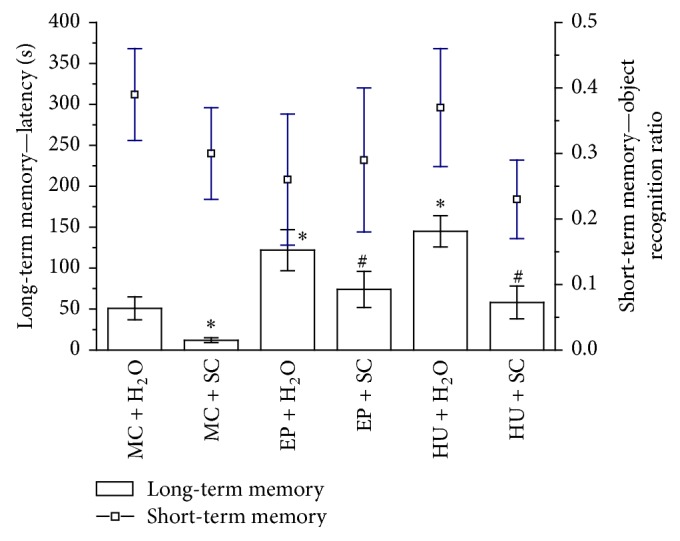
Effect of EP extract (200 mg/kg, p.o.) treatment on short-term and long-term memory in rats. Data are expressed as the means ± SEM. *n*: number of rats. MC + H_2_O: control rats (*n* = 20). MC + SC: scopolamine-treated group (0.5 mg/kg b.w., i.p.) (*n* = 20). EP + H_2_O: extract from root of* Eryngium planum* (*n* = 10). EP + SC: combined extract from root of* Eryngium planum* and scopolamine-treated rats (*n* = 9). HU + H_2_O: Huperzine A treated rats (0.5 mg/kg b.w. p.o.) (*n* = 10). HU + SC: combined Huperzine A and scopolamine-treated rats (*n* = 10). *∗*: versus MC + H_2_O, *P* < 0.05. #: versus MC + SC, *P* < 0.05.

A one-way ANOVA analysis revealed significant differences in long-term memory after using the passive avoidance test (*F*(5,70) = 9.17; *P* < 0.05; Figure 3). The strongest effect leading to an improvement of this paradigm was produced by the EP extract and HU as compared to control rats (EP + H_2_O, *P* < 0.05; HU + H_2_O versus MC + H_2_O, *P* < 0.05). However, SC administration to rats significantly decreased the latency time of passive avoidance task (MC + SC versus MC + H_2_O, *P* < 0.05). After EP or HU combined treatment with SC, an improvement in long-term memory was observed (EP + SC versus MC + S, *P* < 0.05; HU + SC versus MC + SC, *P* < 0.05). Therefore, it can be concluded that administration of the EP extract or HU overcomes the effects shown by SC (Figure 3).

#### 4.2.4. Short-Term Memory

The results of the object recognition test showed that administration of the compounds or the extract did not affect the short-term memory of rats (one-way ANOVA *F*(5,72) = 0.94, *P* < 0.46) (Figure 3).

### 4.3. AChE and BuChE Activity in Rat Brain

**Figure 4:**
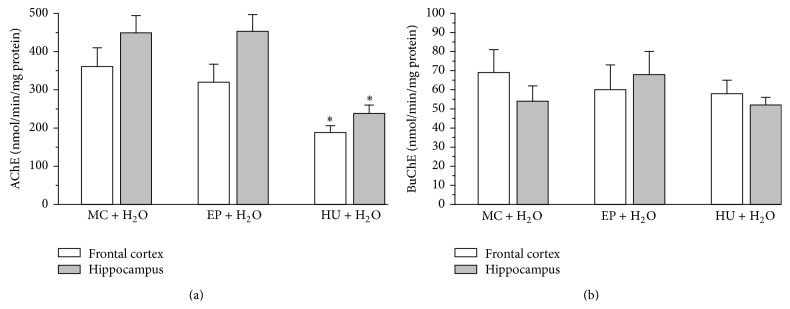
Effect of the EP extract (200 mg/kg, p.o.) on acetylcholinesterase (AChE) (a) and butyrylcholinesterase (BuChE) (b) activities in the frontal cortex or in the hippocampus of rats. Data are expressed as the means ± SEM. *n*: number of rats. MC + H_2_O: control rats (*n* = 9). EP + H_2_O: extract from root of* Eryngium planum* (*n* = 7). HU + H_2_O: Huperzine A treated rats (0.5 mg/kg b.w. p.o.) (*n* = 10). *∗*: versus MC + H_2_O, *P* < 0.05. #: versus MC + SC, *P* < 0.05.

A one-way ANOVA revealed significant differences between the groups in the activity of AChE in both the cortex and the hippocampus (frontal cortex: *F*(2,27) = 4.99, *P* < 0.01; hippocampus: ANOVA *F*(2,23) = 6.20, *P* < 0.05). EP showed an insignificant inhibition of AChE activity in the frontal cortex (only by 11%) as compared to control rats (MC + H_2_O), with no practical action in the hippocampus (Figure 4(a)), whereas HU produced significant inhibition of AChE activity in comparison to the control group, by 48% (*P* < 0.05) and 47% (*P* < 0.05) in the cortex and the hippocampus, respectively. Moreover, there were no significant differences between the values of BuChE activity for EP and HU as compared to the control group (frontal cortex: ANOVA *F*(2,27) = 0.291, *P* > 0.05; hippocampus: ANOVA *F*(2,27) = 1.08, *P* > 0.05) (Figure 4(b)).

### 4.4. AChE, BuChE, and BACE-1 mRNA Expression in Rat Brain

**Figure 5:**
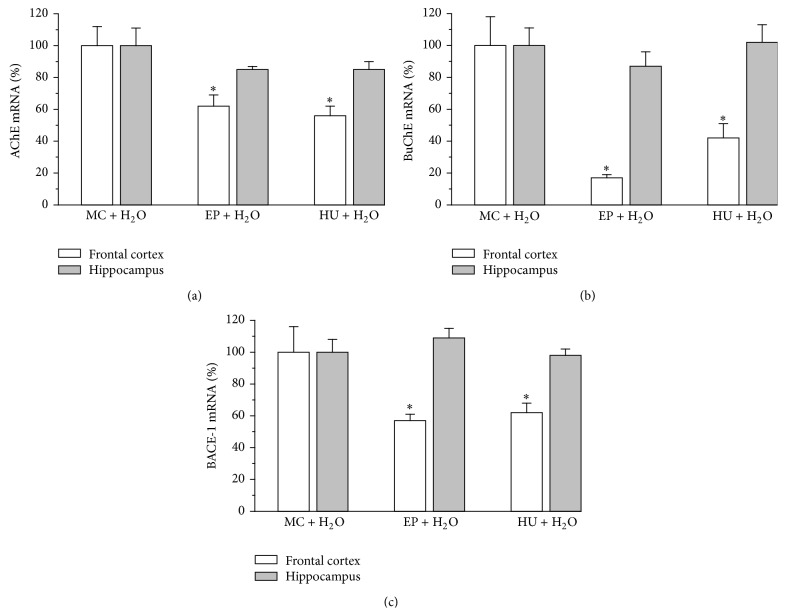
Effect of the EP extract (200 mg/kg, p.o.) on acetylcholinesterase (AChE) (a), butyrylcholinesterase (BuChE) (b), and beta-secretase (BACE-1) (c) mRNA expression levels in the frontal cortex or in the hippocampus of rats. Data are expressed as the means ± SEM. Values expressed as a ratio: the gene/GAPDH. *n*: number of rats. MC + H_2_O: control rats (*n* = 9). EP + H_2_O: extract from root of* Eryngium planum* (*n* = 7). HU + H_2_O: Huperzine A treated rats (0.5 mg/kg b.w. p.o.) (*n* = 10). *∗*: versus MC + H_2_O, *P* < 0.05. #: versus MC + SC, *P* < 0.05.

A one-way ANOVA analysis revealed significant differences of AChE mRNA expression in the cortex (ANOVA *F*(2,23) = 8.03, *P* < 0.05). As shown in Figure 5(a), multiple treatment of EP produced a statistically significant decrease of AChE mRNA level by 38% in the cortex (versus MC + H_2_O, *P* < 0.05), without any effect in the hippocampus (ANOVA *F*(2,26) = 1.62, *P* > 0.05). Similarly, it was demonstrated that administration of HU significantly decreased the relative AChE expression level by 44% in the cortex as compared to the control group (*P* < 0.05), with no effect in the hippocampus. There were also significant differences between the relative values of BuChE mRNA expression (Figure 5(b)), but only in the cortex (frontal cortex: ANOVA *F*(2,19) = 13.31, *P* < 0.05; hippocampus: ANOVA *F*(2,23) = 0.586, *P* > 0.05). Further detailed statistical analysis showed that EP treatment led to a decrease in the BuChE mRNA expression level by 83% in the cortex (versus MC + H_2_O, *P* < 0.05), without any effect on the hippocampus. Prolonged HU administration resulted in a decrease of the transcript level in the cortex by 58% (versus MC + H_2_O). Next analysis revealed significant differences in the BACE-1 mRNA expression in the cortex, but not in the hippocampus (frontal cortex: ANOVA *F*(2,23) = 5.93, *P* < 0.05; hippocampus: ANOVA *F*(2,27) = 0.838, *P* > 0.05) (Figure 5(c)). EP produced a statistically significant decrease of the BACE-1 expression level by 43% in the cortex (versus MC + H_2_O, *P* < 0.05), without any effect on the hippocampus, similarly to HU, as HU treatment led to a decrease in the mRNA expression level by 38% in the cortex (versus MC + H_2_O, *P* < 0.05), but not in the hippocampus.

## 5. Discussion

Recently, a growing amount of data confirmed that numerous flavonoids and phenolic acids, that is, quercetin [39], chlorogenic acid [40], gallic acid [41], and a few plant extracts containing rosmarinic acid and essential oil, that is,* Rosmarinus officinalis* [29, 42], showed antiamnesic effects in Wistar rats. However, it is well known that not only these chemical compounds but also saponins, a diverse group of natural compounds found in a wide variety of plants (i.e., in* Panax ginseng*) and a few marine animals, have been reported to inhibit cholinergic enzymes activity and diminished beta-amyloid deposition [6, 43, 44]. They exert a wide range of pharmacological activities, including expectorant, anti-inflammatory, vasoprotective, immunomodulatory, hypoglycaemic, antifungal, and antiparasitic [45].* Panax ginseng* is the most investigated saponin-rich plant and has been used for medicinal purposes since the antiquity until today. Preclinical and clinical studies have shown that extracts of ginseng leaves, roots, and ginseng saponins have promising therapeutic potential as cognitive enhancing drugs [43, 44, 46, 47]. It should be stressed that the literature lacks reports on their molecular mechanism of action in the CNS. Moreover, a saponin-rich extract from the root of* E. planum* has not been investigated in amnesic animal models. Also, its effect on the activity of AChE and BuChE and secretases (i.e., BACE-1) genes transcription profile in rat brain has not been examined so far. 

*Phytochemical Screening*. Our phytochemical complex analysis showed that the extract from root of* E. planum* contains 64 various chemical constituents. The phytochemical analysis revealed two groups of secondary metabolites present in the* E. planum* roots. The first group included glycosides of hydroxycinnamic acids:* p*-coumaric, ferulic, and caffeic. The caffeic acid is also a component of rosmarinic acid and its derivatives. The polyphenol content was calculated to be equal to 2.45 mg GA per gram of the tissue dry weight. The presence of a high amount of these compounds implicated their significant impact on the holistic biological activity of the EP extract. Saponins with mainly barrigenol skeleton [30] constituted the second and an overwhelming group of secondary metabolites in the EP extract. Also, 36 triterpenoid saponins with undetermined chemical structures were detected in the extract. The biological activity of both groups of metabolites present in the EP extract is well documented in the literature. Their joint and complementary activities may have a positive influence on animal and human health.

According to a previous study [22], the root of* E. planum *does not contain large amounts of essential oils. Hydrodistillation of dried roots gave essential oils in yield of 0.05%. The main components of the root oil were (*Z*)-falcarinol and 2,3,4-trimethylbenzaldehyde; others were monoterpene (limonene, *α*-pinene, and *β*-pinene) and sesquiterpene hydrocarbons. More than 100 constituents were identified according to their retention indices (RI) and mass spectra; (*Z*)-falcarinol (64.4%) was found as the major component of root essential oil. Polyacetylenes such as falcarinol and falcarindiol are widespread among the Apiaceae plant family [22]. 

*Cognitive and Behavioral Tests*. In our study, a model of SC-induced cognitive impairment in rats was used, due to the fact that scopolamine is a muscarinic antagonist that induces central cholinergic blockade, which in turn produces a reversible and well-described impairment in both maintaining attention and processing of information and acquisition of new knowledge in rodents and in humans [48]. This experimental model for AD [49] was used in many other studies coupled with the assessment of cognitive functions in rodents [40, 50, 51].

SC in our study showed an opposite effect in comparison to the extract from* E. planum* roots. Moreover, administration of SC to rats significantly decreased the latency time of passive avoidance task, suggesting that SC impaired the long-term memory in rats (by 76.5% versus MC + H_2_O, control group). The effect seemed to be specific, since SC produced an increase in the spontaneous activity of rats. It is probably due to the fact that low-dose SC used in our study does not act as a CNS depressant and stimulates exploratory behavior in rodents by muscarinic antagonism, facilitating the release of an excitatory neurotransmitter, that is, acetylcholine (turn-over effect), which is sometimes proposed [52]. Moreover, SC administration reduced motor coordination, but it is known that SC demonstrates a lack of correlation between motor skills and learning abilities [53]. 

*AChE and BuChE Activity in Rat Brain*. Additionally, SC was observed to diminish the activity of AChE in the frontal cortex and the hippocampus by 50% and 45%, respectively, and to decrease the activity of BuChE in the frontal cortex and in the hippocampus by 62% and 47%, respectively (data not shown). Analysis of pharmacological results showed that cognitive changes expressed by improvement of long-term (but not short-term) memory after application of EP were similar to HU in both SC-treated and SC-untreated rats. Presumably, the mechanism of action of the extract could be comparable to the reference compound used as a potent antiamnesic agent [54, 55]. However, EP did not inhibit AChE or BuChE activity in the frontal cortex and the hippocampus of rats. Therefore, other mechanisms of action must be taken into account in order to explain the action of the EP extract.

In a study of Wang et al. [47], two major active triterpene saponins from the root of* Panax ginseng* have been shown to exert different mechanism of action in SC-induced dementia mice models. These authors found that saponin Rg1 inhibited AChE activity, while compound Rb1 had no effect on AChE activity. However, both saponins were effective in improving memory deficiency. They also showed that second saponin Rb1 inhibited the decrease level of serotonin (5-HT) induced by scopolamine. Since it is well known that increased brain 5-HT levels have been shown to improve cognitive performance [56, 57], it can be speculated that antiamnestic EP results in our study are coupled with 5-HT. However, at the moment, the question whether multiple EC treatment alters the level of 5-HT remains unanswered. Also Peña et al. [46] observed that although ginsenoside Rg3-enriched fraction did not inhibit acetylcholinesterase activity, it still significantly reversed scopolamine-induced cognitive impairment in mice. Similar activity was observed for soyasaponins Ab and Bb, which prevented scopolamine-induced memory impairment in mice without the inhibition of acetylcholinesterase by increasing BDNF expression and CREB phosphorylation [58]. 

*AChE, BuChE, and BACE-1 mRNA Expression in Rat Brain*. In our study, the EP extract slightly affected the AChE and BuChE mRNAs transcription in the brain homogenates (especially in the frontal cortex) of rats. A 28-day administration of the extract of EP and HU caused a reduction of the activity and AChE mRNA levels in the frontal cortex. Moreover, there was a slight decrease of the BuChE activity, while its transcripts level dropped significantly under the influence of the extract (Figures 4 and 5). The treatment with EP produced a decrease of the relative AChE and BuChE mRNA levels in the cortex by 38% and 83%, respectively. Additionally, EP caused the diminishing of the relative mRNA BACE-1 gene expression level by 43% in the rat cortex, similarly as HU. A different effect of the administrated extract was observed in the hippocampus. There were no important changes in the activity and the levels of AChE and BuChE mRNAs. Their activity was slightly insignificantly elevated, while their mRNA level was slightly reduced as compared to MC + H_2_O (Figures 4 and 5). However, it remains unclear why the diminishing of AChE and BuChE mRNAs does not correlate with the activity of these enzymes. The observed differences in the level of AChE activity and mRNA level can be caused by changes in the activity of AChE in other regions of the brain, not analyzed in this study, such as substantia nigra, cerebellum, globus pallidus, and hypothalamus, where it exerts nonenzymatic neuromodulatory functions affecting neurite outgrowth and synaptogenesis, modulating the activity of the regional cerebral blood flow of other proteins and other functions [59]. Regardless of this, it is difficult to clearly explain why the diminishing of the relative quantities of AChE and BuChE mRNAs did not correlate with the lowering of the activity of these enzymes. Lack of such parallelism was also observed by García-Ayllón et al. [60]. They noted that AChE activity was not paralleled by an increase in mRNA levels. These authors were of the opinion that it was caused by the fact that AChE levels are regulated at transcriptional, posttranscriptional, and posttranslational levels, leading to complex expression patterns which can be modulated by physiological and pathological conditions. However, these mechanisms are not fully understood and further studies are needed. Additionally, the mechanism underlying the reversal of SC-induced amnesia by the saponin-rich EP extract remains to be fully elucidated but is not related to the inhibition of AChE and BuChE activity. Future studies should be also performed in order to explain the influence of the EP extract on 5-HT, other neurotransmitter levels, or neurotrophic factors implicated in the improvement of long-term memory.

## 6. Conclusions

There is a possible positive relationship between EP-induced enhancement of long-term memory and the inhibitory effect on mRNA expression levels of AChE, BuACh, and BACE-1 in the frontal cortex of rats. It seems that EP partially acts* via* the cholinergic pathway; however the mechanisms of EP action are probably more complex, since its role as a modulator of beta-secretase activity (due to inhibition of BACE-1 mRNA transcription in frontal cortex) should be taken into consideration. Our results indicate that EP may express neuroprotective activity on some characteristic features of AD, but a detailed chemical composition of EP and its potential role in AD prevention need to be further studied. The above-mentioned results suggest that the pharmacological actions of the ethanolic extract of* Eryngii plani *radixmay be attributed, in part, to its total saponin content. To the best of our knowledge, our study has been the first to evaluate the antiamnestic effect of the root extract of* Eryngium* species. However, further investigations are necessary to establish the neurochemical mechanism of action for this extract in more detail.

## Figures and Tables

**Table 1:** Metabolites detected in the EP extract by HPLC-UV-MS.

Number	RT [min]	MW	Fragmentation in negative ion mode ESI(−)	Fragmentation in positive ion mode ESI(+)	Maximum of absorbance [nm]	Compound identification	Identification level^a^	References
1	6,5	187		188, 146	275	Tyrosine	1	std
2	7,7	204		206, 166	280	Tryptophan	1	std
3	8,6	490		491, 329, 149, 147	270	*p*-Coumaric acid diglucoside	3	[36]
4	9,9	296		297, 147	280sh	*p*-Coumaric acid pentoside	3	[36]
5	9	552	389, 193		280sh	Ferulic acid derivative	3	[20]
6	11	666	665, 503, 252, 161		270	Caffeic acid, triglucoside	3	[36]
7	13,6	342	341, 161, 119		285	Caffeic acid, glucoside	2	[36]
8	14,4	474	473, 359, 179, 161		280, 300	Rosmarinic acid, pentoside	3	[17]
9	15	846	847, 845, 685, 523, 361, 341		285, 305sh	Rosmarinic acid, triglucoside	3	[17]
10	15,4	684	683, 521, 359, 197		280, 320	Rosmarinic acid diglucoside	3	[17]
11	16	656	655, 493, 331, 323, 161		280, 305	Caffeoyl-trihydroxybenzoic acid, diglucoside	3	[20]
12	17,3	698	697, 637, 475, 397		275	Unidentified		
13	19,6	522	521, 359, 197, 179, 161		325sh	Rosmarinic acid, glucoside	2	[17]
14	21,5	1284	1283, 1121, 959, 797, 635, 473		250	Triterpenoid saponin		
15	22	1138	1137, 975, 813, 651, 567		250	Triterpenoid saponin		
16	22,6	1136	1135, 925, 763, 601, 439		260	Triterpenoid saponin		
17	23,9	360	359, 197, 179		320sh	Rosmarinic acid	2	[17]
18	24,6	536	535, 359, 197, 179		320sh	Rosmarinic acid, glucuronide	3	[17]
19	25,1	974	973, 925, 811, 763, 613, 537, 455, 423		280	Triterpenoid saponin		
20	25,4	1180	1179, 1017, 855, 765, 651, 604		275	Triterpenoid saponin		
21	25,8	1122	1121, 959, 797, 635, 473, 423, 409		280	Triterpenoid saponin		
22	26,8	844	843, 663, 573		275	Triterpenoid saponin		
23	27,3	992	991, 829, 667, 618, 455		275	Triterpenoid saponin		
24	27,6	1106	1105, 943, 781, 619		280	Triterpenoid saponin		
25	28,5	886	885, 723, 615, 555, 405		280	Hex-triterpenoid acid		
26	28,9	976	975, 925, 813, 651, 567, 489, 439		270	Barringtogenol C, triglucoside	3	[37]
27	30,3	1252	1251, 1089, 751, 927, 589		230	triterpenoid saponin	3	[30]
28	31,3	1092	1091, 959, 797, 635, 580			Triterpenoid saponin		
29	31,5	1120	1119, 957, 909, 747, 585		230, 280	Triterpenoid saponin	3	[30]
30	31,7	342	341, 179		305	Decaffeinate	3	[20]
31	31,5	968	967, 805, 787, 697		230, 280	3-O-*β*-D-Glucopyranosyl-(1→2)-*β*-d-glucuronopyranosyl-21-O-acetyl-22-O-angeloyl-R1-barrigenol II isomer	2	[30]
32	32	1104	1103, 1085, 1041, 865, 833, 703, 567		230, 280	Triterpenoid saponin	3	[30]
33	32,5	1090	1089, 927, 751, 589		280	Triterpenoid saponin	3	[30]
34	33	958	957, 795, 751, 589		280	Triterpenoid saponin		
35	33,1	942	941, 809, 779, 671, 601, 557, 491, 389		280	Triterpenoid saponin		
36	33,5	1266	1265, 1103, 941, 779, 617, 409		280	Triterpenoid saponin		
37	33,7	1016	1015, 967, 853, 805, 643, 583			Triterpenoid saponin		
38	33,9	1222	1221, 1059, 897, 736, 487		280	Triterpenoid saponin		
39	34,5	942	941, 779, 617		280	Triterpenoid saponin		
40	34,7	926	925, 795, 825, 655, 556, 437		230, 280	3-O-*β*-D-Glucopyranosyl-(1→2)-*β*-D-glucuronopyranosyl-22-O-angeloyl-R1-barrigenol		
41	35,2	956	955, 793, 749, 613, 453		280	Hex-Hex-triterpenoid acid	3	[30]
42	34,9	926	925, 809, 655, 539		280	Triterpenoid saponin	2	[30]
43	35,1	960	959, 797, 633		280	Hex-Rham-triterpenoid acid		
44	35,6	984	983, 851, 713, 643		280	Triterpenoid saponin		
45	36	968	967, 867, 697, 627		280	3-O-*β*-D-Glucopyranosyl-(1→2)-*β*-d-glucuronopyranosyl-21-O-acetyl-22-O-angeloyl-R1-barrigenol II isomer	2	[30]
46	36,3	910	909, 809, 639, 539, 509		280	3-O-*β*-D-Glucopyranosyl-(1→2)-*β*-D-glucuronopyranosyl-22-O-angeloyl-A1-barrigenol	2	[30]
47	37,4	908	907, 727, 637, 567		280	Triterpenoid saponin	3	[30]
48	38,1	766	765, 655, 427, 383, 341, 323		280	Triterpenoid saponin		
49	38,2	970	969, 867, 787, 699, 629		280	Triterpenoid saponin		
50	38,3	694	693, 651, 633, 489, 471, 327, 293, 221		280	Acetyl-glucoside triterpenoid		
51	38,4	1084		1085, 907, 718, 574	280	Triterpenoid saponin		
52	38,5	936	935, 665, 596		280	Triterpenoid saponin		
53	38,6	894	893, 729, 603, 569, 625		230	Triterpenoid saponin		
54	39,7	952	951, 681, 611		230	Triterpenoid saponin		
55	39,7	954	953, 683, 581		230	Triterpenoid saponin	3	[30]
56	40,4	996	995, 797, 751, 629		230	Triterpenoid saponin		
57	40,5	938	937, 739, 693, 657, 571, 489, 423		290	Triterpenoid saponin		
58	40,7	636	635, 473, 424, 311, 161		270, 290	Glucoside triterpenoid		
59	41,2	1154	1153, 991, 829, 667, 599		270	Barringenol R1-tetraglucoside	3	[37]
60	41,4	618	677, 617, 455, 321		270, 275	Betulinic acid glucoside		
61	41,7	1010	1009, 925, 764, 601		250	Triterpenoid saponin		
62	41,8	844	843, 573, 505, 489		280	Triterpenoid saponin	3	[30]
63	42	1178	1177, 1015, 853, 691, 599		280	Triterpenoid saponin		
64	42,1	828	827, 647, 557, 477		280	Triterpenoid saponin	3	[30]

^a^Metabolite identification level according to the Metabolomics Standards Initiative recommendation [38]; rt: retention tome; mw: molecular weight.

## References

[1] Davinelli S., Sapere N., Zella D., Bracale R., Intrieri M., Scapagnini G. (2012). Pleiotropic protective effects of phytochemicals in Alzheimer's disease. *Oxidative Medicine and Cellular Longevity*.

[2] Gomes N. G. M., Campos M. G., Órfão J. M. C., Ribeiro C. A. F. (2009). Plants with neurobiological activity as potential targets for drug discovery. *Progress in Neuro-Psychopharmacology & Biological Psychiatry*.

[3] Kadiyala M., Ponnusankar S., Elango K. (2014). Screening of siddha medicinal plants for its in-vitro acetylcholinesterase and butyrylcholinesterase inhibitory activity. *Pharmacognosy Magazine*.

[4] Mathew M., Subramanian S. (2014). *In vitro* screening for anti-cholinesterase and antioxidant activity of methanolic extracts of ayurvedic medicinal plants used for cognitive disorders. *PLoS ONE*.

[5] Carpinella M. C., Andrione D. G., Ruiz G., Palacios S. M. (2010). Screening for acetylcholinesterase inhibitory activity in plant extracts from Argentina. *Phytotherapy Research*.

[6] Mukherjee P. K., Kumar V., Mal M., Houghton P. J. (2007). Acetylcholinesterase inhibitors from plants. *Phytomedicine*.

[7] Craig L. A., Hong N. S., McDonald R. J. (2011). Revisiting the cholinergic hypothesis in the development of Alzheimer's disease. *Neuroscience & Biobehavioral Reviews*.

[8] Chaiyana W., Okonogi S. (2012). Inhibition of cholinesterase by essential oil from food plant. *Phytomedicine*.

[9] Goedert M., Spillantini M. G. (2006). A century of Alzheimer's disease. *Science*.

[10] Adams M., Gmünder F., Hamburger M. (2007). Plants traditionally used in age related brain disorders–a survey of ethnobotanical literature. *Journal of Ethnopharmacology*.

[11] Vauzour D. (2012). Dietary polyphenols as modulators of brain functions: biological actions and molecular mechanisms underpinning their beneficial effects. *Oxidative Medicine and Cellular Longevity*.

[12] Zhang H. Y., Zheng C. Y., Yan H. (2008). Potential therapeutic targets of huperzine A for Alzheimer's disease and vascular dementia. *Chemico-Biological Interactions*.

[13] Keller C., Kadir A., Forsberg A., Porras O., Nordberg A. (2011). Long-term effects of galantamine treatment on brain functional activities as measured by pet in Alzheimer's disease patients. *Journal of Alzheimer's Disease*.

[14] Choi S.-H., Hur J.-M., Yang E.-J. (2008). Beta-secretase (BACE 1) inhibitors from *Perilla frutescens* var. *acuta*. *Archives of Pharmacal Research*.

[15] Kim D. S. H. L., Kim J.-Y., Han Y. S. (2007). Alzheimer's disease drug discovery from herbs: neuroprotectivity from *β*-amyloid (1–42) insult. *Journal of Alternative and Complementary Medicine*.

[16] Wang P., Su Z., Yuan W., Deng G., Li S. (2012). Phytochemical constituents and pharmacological activities of *Eryngium* L. (Apiaceae). *Pharmaceutical Crops*.

[17] Claire E. L., Schwaiger S., Banaigs B., Stuppner H., Gafner F. (2005). Distribution of a new rosmarinic acid derivative in *Eryngium alpinum* L. and other Apiaceae. *Journal of Agricultural and Food Chemistry*.

[18] Hohmann J., Páll Z., Günther G., Máthé I. (1997). Flavonolacyl glycosides of the aerial parts of *Eryngium campestre*. *Planta Medica*.

[19] Kholkhal W., Ilias F., Bekhechi C., Bekkara F. (2012). *Eryngium maritimum*: a rich medicinal plant of polyphenols and flavonoids compounds with antioxidant, antibacterial and antifungal activities. *Current Research Journal of Biological Sciences*.

[20] Zhang Z. Z., Li S. Y., Ownby S. (2008). Phenolic compounds and rare polyhydroxylated triterpenoid saponins from *Eryngium yuccifolium*. *Phytochemistry*.

[21] Erdelmeier C. A. J., Sticher O. (1985). Coumarin derivatives from *Eryngium campestre*. *Planta Medica*.

[22] Thiem B., Kikowska M., Kurowska A., Kalemba D. (2011). Essential oil composition of the different parts and *In Vitro* shoot culture of *Eryngium planum* L.. *Molecules*.

[23] Christensen L. P., Brandt K. (2006). Bioactive polyacetylenes in food plants of the Apiaceae family: occurrence, bioactivity and analysis. *Journal of Pharmaceutical and Biomedical Analysis*.

[24] Küpeli E., Kartal M., Aslan S., Yesilada E. (2006). Comparative evaluation of the anti-inflammatory and antinociceptive activity of Turkish *Eryngium* species. *Journal of Ethnopharmacology*.

[25] Thiem B., Goslińska O., Kikowska M., Budzianowski J. (2010). Antimicrobial activity of three *Eryngium* L. species (*Apiaceae*). *Herba Polonica*.

[26] Cádiz-Gurrea M. D. L. L., Fernández-Arroyo S., Joven J., Segura-Carretero A. (2013). Comprehensive characterization by UHPLC-ESI-Q-TOF-MS from an *Eryngium bourgatii* extract and their antioxidant and anti-inflammatory activities. *Food Research International*.

[27] Thiem B., Kikowska M., Krawczyk A., Wieckowska B., Sliwinska E. (2013). Phenolic acid and DNA contents of micropropagated *Eryngium planum* L. *Plant Cell, Tissue and Organ Culture*.

[28] Wojakowska A., Piasecka A., García-López P. M. (2013). Structural analysis and profiling of phenolic secondary metabolites of Mexican lupine species using LC-MS techniques. *Phytochemistry*.

[29] Ozarowski M., Mikolajczak P. L., Bogacz A. (2013). *Rosmarinus officinalis* L. leaf extract improves memory impairment and affects acetylcholinesterase and butyrylcholinesterase activities in rat brain. *Fitoterapia*.

[30] Kowalczyk M., Masullo M., Thiem B., Piacente S., Stochmal A., Oleszek W. (2014). Three new triterpene saponins from roots of *Eryngium planum*. *Natural Product Research*.

[31] Cheng C. M., Jalil A. M. M., Ismail A. (2009). Phenolic and theobromine contents of commercial dark, milk and white chocolates on the Malaysian market. *Molecules*.

[32] Boissier P. J. R., Tardy J., Diverres J. C. (1960). Une nouvelle méthode simple pour explorer l'action ‘tranquillisante’: le test de la cheminée. *Medicina Experimentalis*.

[33] Ader R., Weijnen J. A. W. M., Moleman P. (1972). Retention of a passive avoidance response as a function of the intensity and duration of electric shock. *Psychonomic Science*.

[34] Blalock E. M., Chen K.-C., Sharrow K. (2003). Gene microarrays in hippocampal aging: statistical profiling identifies novel processes correlated with cognitive impairment. *Journal of Neuroscience*.

[35] Isomae K., Morimoto S., Hasegawa H., Morita K., Kamei J. (2003). Effects of T-82, a novel acetylcholinesterase inhibitor, on impaired learning and memory in passive avoidance task in rats. *European Journal of Pharmacology*.

[36] Parveen I., Wilson T., Donnison I. S., Cookson A. R., Hauck B., Threadgill M. D. (2013). Potential sources of high value chemicals from leaves, stems and flowers of *Miscanthus sinensis* ‘Goliath’ and *Miscanthus sacchariflorus*. *Phytochemistry*.

[37] Hiller K., Nguyen V. T., Lehman G., Grundemann E. (1974). Saniculoidae components. 21. Betulinic acid, a sapogenin in *Eryngium bromelifolium*. *Pharmazie*.

[38] Sumner L. W., Amberg A., Barrett D. (2007). Proposed minimum reporting standards for chemical analysis working group (CAWG) metabolomics standards initiative (MSI). *Metabolomics*.

[39] Bhutada P., Mundhada Y., Bansod K. (2010). Ameliorative effect of quercetin on memory dysfunction in streptozotocin-induced diabetic rats. *Neurobiology of Learning and Memory*.

[40] Kwon S.-H., Lee H.-K., Kim J.-A. (2010). Neuroprotective effects of chlorogenic acid on scopolamine-induced amnesia via anti-acetylcholinesterase and anti-oxidative activities in mice. *European Journal of Pharmacology*.

[41] Nagpal K., Singh S. K., Mishra D. N. (2013). Nanoparticle mediated brain targeted delivery of gallic acid: in vivo behavioral and biochemical studies for protection against scopolamine-induced amnesia. *Drug Delivery*.

[42] Zanella C. A., Treichel H., Cansian R. L., Roman S. S. (2012). The effects of acute administration of the hydroalcoholic extract of rosemary (*Rosmarinus officinalis* L.) (Lamiaceae) in animal models of memory. *Brazilian Journal of Pharmaceutical Sciences*.

[43] Wang C.-M., Liu M.-Y., Wang F. (2013). Anti-amnesic effect of pseudoginsenoside-F11 in two mouse models of Alzheimer's disease. *Pharmacology Biochemistry and Behavior*.

[44] Kim E.-J., Jung I.-H., Van Le T. K., Jeong J.-J., Kim N.-J., Kim D.-H. (2013). Ginsenosides Rg5 and Rh3 protect scopolamine-induced memory deficits in mice. *Journal of Ethnopharmacology*.

[45] Sparg S. G., Light M. E., van Staden J. (2004). Biological activities and distribution of plant saponins. *Journal of Ethnopharmacology*.

[46] Peña I. D., Yoon S. Y., Kim H. J. (2014). Effects of ginseol k-g3, an Rg3-enriched fraction, on scopolamine-induced memory impairment and learning deficit in mice. *Journal of Ginseng Research*.

[47] Wang Q., Sun L.-H., Jia W. (2010). Comparison of ginsenosides Rg1 and Rb1 for their effects on improving scopolamine-induced learning and memory impairment in mice. *Phytotherapy Research*.

[48] Yamada N., Hattori A., Hayashi T., Nishikawa T., Fukuda H., Fujino T. (2004). Improvement of scopolamine-induced memory impairment by Z-ajoene in the water maze in mice. *Pharmacology Biochemistry and Behavior*.

[49] Ebert U., Kirch W. (1998). Scopolamine model of dementia: electroencephalogram findings and cognitive performance. *European Journal of Clinical Investigation*.

[50] Jain S., Sangma T., Shukla S. K., Mediratta P. K. (2015). Effect of *Cinnamomum zeylanicum* extract on scopolamine-induced cognitive impairment and oxidative stress in rats. *Nutritional Neuroscience*.

[51] Orhan I., Aslan M. (2009). Appraisal of scopolamine-induced antiamnesic effect in mice and in vitro antiacetylcholinesterase and antioxidant activities of some traditionally used Lamiaceae plants. *Journal of Ethnopharmacology*.

[52] Vohora D., Pal S. N., Pillai K. K. (2000). Effect of locomotor activity on the passive avoidance test for the evaluation of cognitive function. *Indian Journal of Pharmacology*.

[53] Thouvarecq R., Protais P., Jouen F., Caston J. (2001). Influence of cholinergic system on motor learning during aging in mice. *Behavioural Brain Research*.

[54] Ha G. T., Wong R. K., Zhang Y. (2011). Huperzine a as potential treatment of Alzheimer's disease: an assessment on chemistry, pharmacology, and clinical studies. *Chemistry & Biodiversity*.

[55] Tun M. K. M., Herzon S. B. (2012). The pharmacology and therapeutic potential of (−)-huperzine A. *Journal of Experimental Pharmacology*.

[56] Essman W. B. (1974). Brain 5-hydroxytryptamine and memory consolidation. *Advances in Biochemical Psychopharmacolog*.

[57] Olivier J. D. A., Jans L. A. W., Blokland A. (2009). Serotonin transporter deficiency in rats contributes to impaired object memory. *Genes, Brain and Behavior*.

[58] Hong S.-W., Yoo D.-H., Woo J.-Y., Jeong J.-J., Yang J.-H., Kim D.-H. (2014). Soyasaponins Ab and Bb prevent scopolamine-induced memory impairment in mice without the inhibition of acetylcholinesterase. *Journal of Agricultural and Food Chemistry*.

[59] Papandreou M. A., Dimakopoulou A., Linardaki Z. I. (2009). Effect of a polyphenol-rich wild blueberry extract on cognitive performance of mice, brain antioxidant markers and acetylcholinesterase activity. *Behavioural Brain Research*.

[60] García-Ayllón M.-S., Cauli O., Silveyra M.-X. (2008). Brain cholinergic impairment in liver failure. *Brain*.

